# Prevention of Anthracycline-Induced Cardiotoxicity: The Good and Bad of Current and Alternative Therapies

**DOI:** 10.3389/fcvm.2022.907266

**Published:** 2022-06-22

**Authors:** Nonhlakanipho F. Sangweni, Derick van Vuuren, Lawrence Mabasa, Kwazi Gabuza, Barbara Huisamen, Sharnay Naidoo, Reenen Barry, Rabia Johnson

**Affiliations:** ^1^Biomedical Research and Innovation Platform, South African Medical Research Council, Cape Town, South Africa; ^2^Division of Medical Physiology, Faculty of Medicine and Health Sciences, Centre for Cardio-metabolic Research in Africa, Stellenbosch University, Stellenbosch, South Africa; ^3^Research and Development Department, BioPharm, Hamilton, New Zealand

**Keywords:** cardiotoxicity, doxorubicin, flavonoids, cardioprotection, drug-drug interaction

## Abstract

Doxorubicin (Dox)-induced cardiotoxicity (DIC) remains a serious health burden, especially in developing countries. Unfortunately, the high cost of current preventative strategies has marginalized numerous cancer patients because of socio-economic factors. In addition, the efficacy of these strategies, without reducing the chemotherapeutic properties of Dox, is frequently questioned. These limitations have widened the gap and necessity for alternative medicines, like flavonoids, to be investigated. However, new therapeutics may also present their own shortcomings, ruling out the idea of “natural is safe”. The U.S. Food and Drug Administration (FDA) has stipulated that the concept of drug-safety be considered in all pre-clinical and clinical studies, to explore the pharmacokinetics and potential interactions of the drugs being investigated. As such our studies on flavonoids, as cardio-protectants against DIC, have been centered around cardiac and cancer models, to ensure that the efficacy of Dox is preserved. Our findings thus far suggest that flavonoids of *Galenia africana* could be suitable candidates for the prevention of DIC. However, this still requires further investigation, which would focus on drug-interactions as well as *in vivo* experimental models to determine the extent of cardioprotection.

## Introduction

Over the years much effort has been placed on understanding the molecular and cellular biology of numerous cancers, which has led to rapid progressions in diagnostics, drug discovery and prevention of cancer-related morbidities and mortalities (1). In modern oncology, the introduction of chemotherapeutic regimens has been identified as a major contributing factor to the observed increased life expectancy of cancer patients. Notably, today, more than 67% of adult cancer patients will live up to 5 years after diagnosis, and over 75% of pediatric cancer patients will have a 10 year survival rate after diagnosis (2). Generally, chemotherapeutic drugs work by targeting cells at different phases of the cell cycle, which aids in predicting which drugs are likely to work well-together or be effective for a specific cancer (3). However, researchers have found that while chemotherapeutics were designed to target mutated and rapidly dividing cells, these drugs are unable to discern between cancer and healthy cells (4, 5). Therefore, normal cells, which can trigger a self-initiated healing response, are also damaged, and eradicated during chemotherapy. Literature indicates that chemotherapeutic drugs, like anthracyclines (ATCs), are associated with a higher incidence of inducing cardiotoxic effects, which may progress into organ failure, relative to other cancer therapies (2, 6, 7). Therefore, the potent efficacy of ATCs is often overshadowed by their cardiotoxic side effects, which has limited their clinical use (8). Numerous studies investigating the mechanisms and risks associated with ATC-induced cardiotoxicity (AIC) have been conducted using doxorubicin (Dox) as a representative chemotherapeutic drug. Thus, the current review was formulated with a focus on Dox to discuss the incidence of cancer mortalities and risks associated with ATC-induced cardiotoxicity (AIC). Additionally, we also discuss the use of alternative therapies against AIC and how they may influence the pharmacology of Dox.

## Incidence of Cancer-Related Deaths

**Figure 1:**
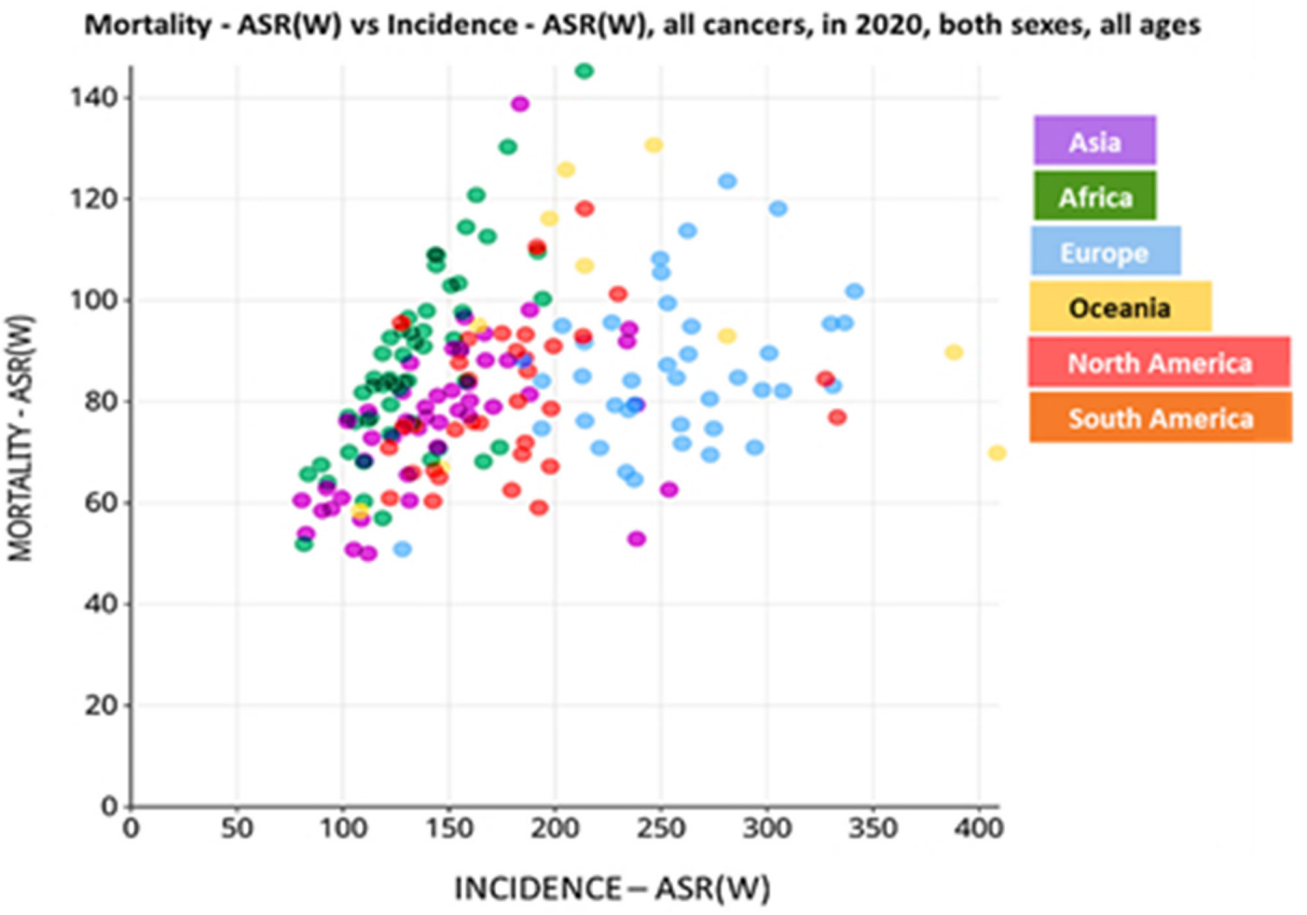
Incidence of cancer-related deaths in the different continents, per 100,000 population. Data is based on the incidence vs. mortality rate of cancer patients in the year 2020. World age-standardized rate [ASR (W)]. Data source: Globocan 2020; World Health Organization (WHO) 2021.

Approximately 10 million cancer-related deaths were recorded in 2020, making cancer the second leading cause of death worldwide (9). In Africa, the gruesome disparities that exist between public and private health sector's, which are driven by socio-economic factors, is directly reflected by the high cancer mortality rate vs. cancer incidence (Figure 1) (10). In contrast, most people in first-world regions, like America, have access to health insurance and therefore, present with a decreased cancer mortality rate vs. incidence when compared to the African and Asian communities (Figure 1) (10). To fully understand the global impact of cancer and progress made thereof, the American Cancer Society (ACS) reports that quantifying cancer-related mortalities, which are unlikely to be influenced by new diagnoses and survival outcomes within populations, can provide better insight into the disease burden (11).

**Figure 2:**
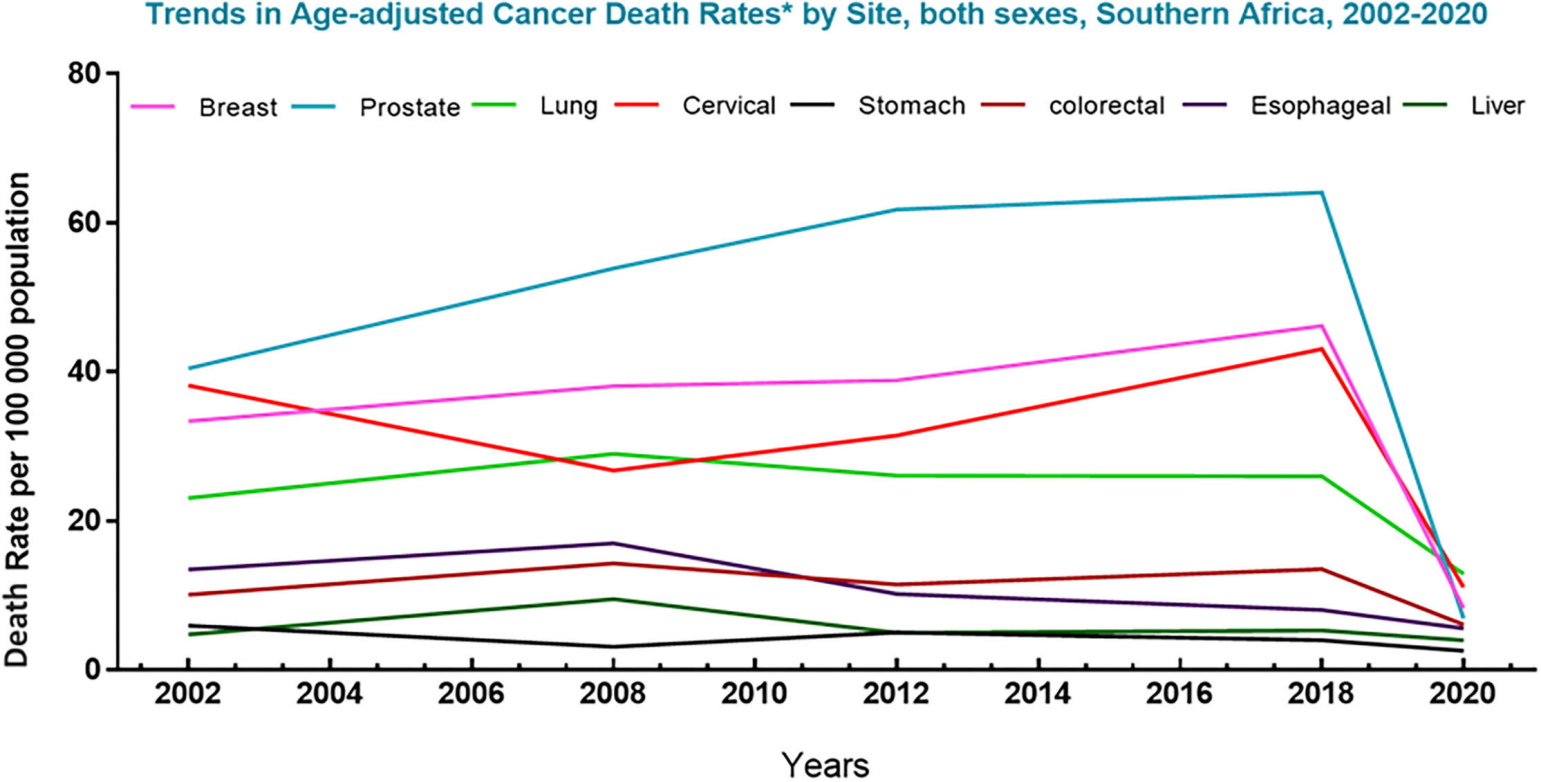
Trends in cancer mortality in Southern Africa (2002–2020). Graph represents deaths associated with the top 8 cancers in South Africa for men and women, per 100,000 population, over the last decades. The data shows a sharp decline in the death rate of the top eight cancers in this region. Data source: Globocan 2021; Hamdi et al. (13).

In regions like South Africa platforms like the National Cancer Registry (NCR), which were formulated to provide data on the burden of cancer in both developing and developed countries with the aim of creating global awareness, remains poorly sourced and outdated (12). In this region, the registry was last updated in 2017 using data acquired from cancer deaths recorded in 2014. Such shortfalls and inconsistencies in data capturing make it difficult to efficiently track and manage the incidence of cancer and its co-morbidities in these demographics. Evidently, a planned population-based registry is clinically fundamental to drive decisions involving the screening and prevention of cancers, as well as the development of cancer treatment. Notably, since the early 1960's, the ACS has reported an increasing trend in the 5-year survival rate of cancer from 27 to 63%, for African patients, and from 39 to 68%, for Caucasian patients (11). In Southern Africa, an 86.9% survival increase in patients with the top eight cancers was reported from 2002 to 2020 (Figure 2) (10, 14). This improvement was largely driven by developments made against the top four cancers (breast, prostate, cervical and lung) in this demographic and the advances in chemotherapeutic agents, such as Dox.

## Pharmacology of Doxorubicin

**Figure 3:**
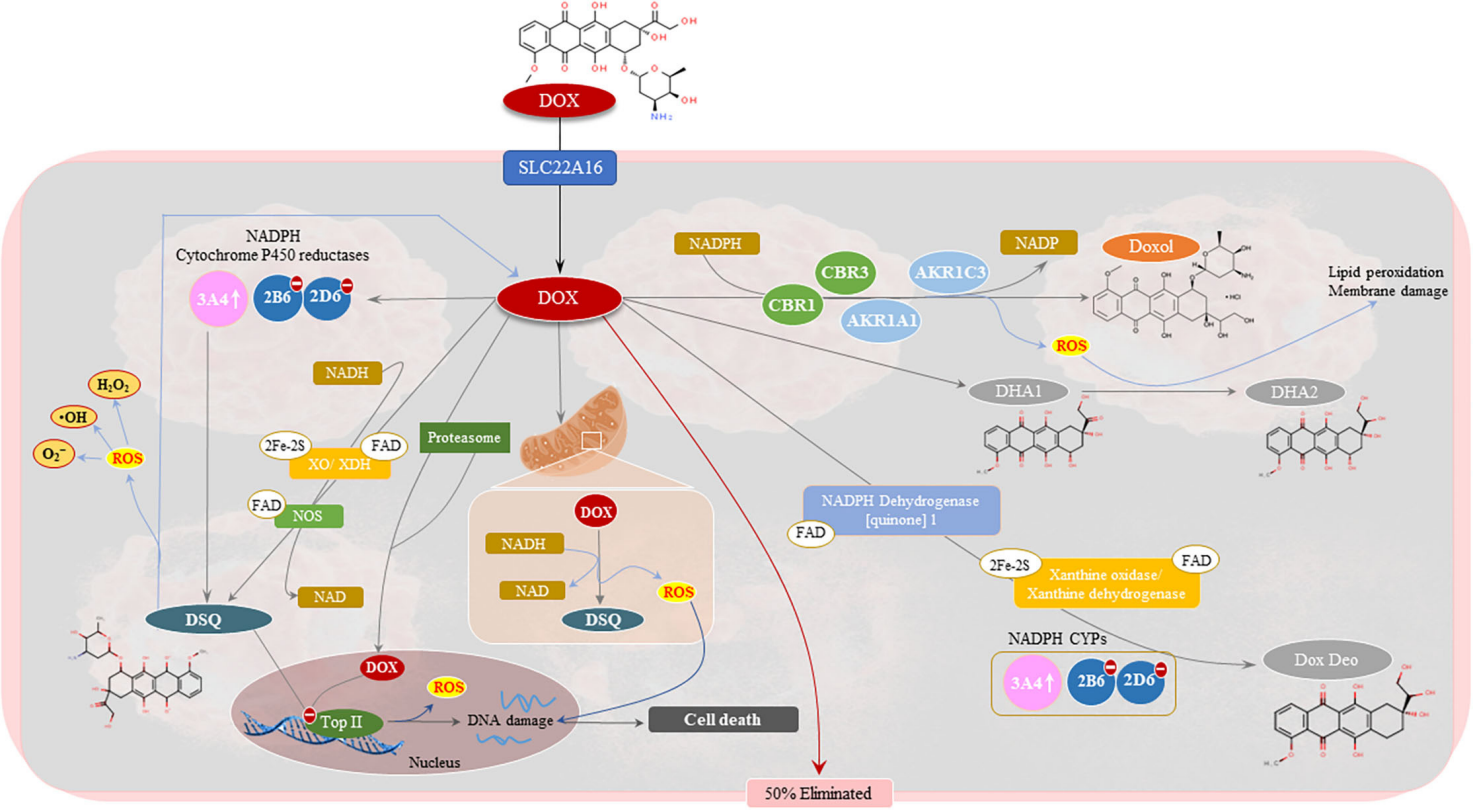
Pharmacology of doxorubicin (Dox). Approximately 50% of Dox is eliminated from the body unchanged. The remaining Dox undergoes three metabolic processes to form doxorubicinol (Doxol), semiquinone radicals (DSQ), and 7-deoxyaglycone and hydroxyaglycone, respectively. The two-electron reduction of Dox forms Doxol *via* several oxidoreductases namely, carbonyl reductase 1 (CBR1) and 3 (CBR3), and aldo-keto reductases family 1 member (AKR1C3) and AKR1A1, in the presence of nicotinamide adenine dinucleotide phosphate (NADPH). Semiquinone formation entails the one-electron reduction of Dox and is mediated by mitochondrial and cytosolic NADPH dehydrogenates, xanthine oxidase (XO)/dehydrogenase (XDH), NADPH cytochrome P450 (CYPs) reductases and nitric oxide synthases (NOS). Deglycosidation of Dox, in the presence of NADPH-CYPs, XO/XDH and NADPH dehydrogenase. The formation of these metabolites is associated with DNA damage, *via* topoisomerase II inhibition, and the production of hydroxyl radicals (•OH), superoxide anions (${\text{O}}_{2}^{-}$) and peroxides (H_2_O_2_). This results in the activation of tumoricidal pathways which drive cancer cell death.

The anticancer properties of Dox can be linked to the presence of flat aromatic moieties in Dox which allows it to form complexes with DNA by intercalating between the DNA base-pairs thereby, causing bidirectional transmission of positive torsion (15). The latter impedes topoisomerase II alpha (Top II-α) activity, which is needed for the regulation of DNA's super-helical state and unlinking intertwined DNA strands (16). Inhibition of Top II by Dox, stabilizes the DNA-Top II complex which disrupts the religation portion of the ligation-religation reaction (15). This results in DNA double-stranded breaks (DSBs) and fragmented nuclei with condensed chromatin, which triggers cancer cell death pathways such as apoptosis and necrosis (16). Also contributing to its tumoricidal and anti-carcinogenic properties, is Dox's ability to induce oxidative damage which is driven by the reduction of Dox to its secondary metabolites [doxorubicinol (Doxol), semiquinones (DSQ) and aglycones], a reaction catalyzed by NADPH cytochrome P450 (CYP) and carbonyl reductases (CBRs) (16–18). During its metabolism, the C-13 carbonyl group of Dox is reduced by CBR1 and CBR3 to Doxol, which then undergoes acid-catalyzed hydrolysis and then protonation at C-7 to form a double reduced C7-deoxyaglycone (16). C7-quinone-methide, which is a tautomer of C7-deoxyaglycone, generates reactive oxygen species (ROS) by covalently binding to DNA. Similarly, the production of Dox-semiquinones, *via* NADPH CYP enzymes, triggers oxidative stress-induced damage by generating superoxide's (${\text{O}}_{2}^{-}$), hydroxyl radicals (•OH) and peroxides (H_2_O_2_), which causes further DNA damage (16) thereby, accelerating cancer apoptosis and aiding in combatting cancer (Figure 3). The metabolism of Dox can be further driven by mitochondrial and cytosolic NADPH dehydrogenases, xanthine oxidase (XO) or dehydrogenase (XDH), and nitric oxide synthases (NOS), to form more DSQs and aglycones. The increase in these metabolites in the circulatory system, has been associated with the occurrence of adverse reactions, like cardiotoxicity (19, 20). However, literature notes that while these metabolites induce cardiotoxicity more potently than their parent compound, Dox, these metabolites are not as effective at combatting cancer than Dox (21).

## DIC: Are Today's Cancer Survivors the Future CVD Patients

Despite being a first-line anti-cancer drug, the clinical use of Dox has been surrounded by much controversy. On the one hand, the dramatic developments in chemotherapeutic drugs have increased the life expectancy of cancer patients, with the number of survivors projected to rise exponentially in the coming years (22). On the other hand, cancer survivors present with an increased cardiovascular disease (CVD) risk and are expected to develop cardiomyopathies and metabolic toxicities within months or years after treatment cessation, consequently exacerbating the burden of CVDs (1). However, since chemotherapy has prolonged and enhanced the quality of life, the risk of cardiotoxicity is often outweighed by the overall benefit of cancer treatment. Generally, the risk of developing Dox-induced cardiomyopathy (DIC) is escalated as the cumulative dose of Dox increases: where a dose of 400 mg/m^2^ increases the risk of DIC by 3–5% and that of 700 mg/m^2^ effectuates an 18–48% DIC risk (Table 1) (8). As a result, oncologists caution that the cumulative dose of Dox should be limited to ≤ 550 mg/m^2^ (2). However, even at relatively lower doses, the risk of developing cardiotoxicity is still present, especially amongst pediatric survivors (23). Evidently, children between the ages of 0–4 years old, who received lower ATC doses (1–249 mg/m^2^) presented with an increased incidence of cardiomyopathy relative to children who were exposed to ATCs at an age of older than 13. In addition, higher ATC doses (≥250 mg/m^2^) led to an even higher risk of DIC in both 0–4 years old [relative rate (RR), 4.0; 95% cumulative incidence (CI), 2.5–6.4]and 4–13 years old kids (RR, 2.4; 95% CI, 1.7–3.5) at diagnosis when compared to kids older than 13 years (24). Thus, reiterating that younger individuals and patients receiving higher Dox doses have an increased predisposition to DIC.

**Table 1:** Factors associated with increased risk of DIC.

**Cumulative dose and predictive risk**		**Other risks**
150 mg/m^2^	0.2%	Females
300 mg/m^2^	1.6%	Children ≤ 4 years receiving low ATC dose Children ≥ 13 years old receiving high ATC dose
400 mg/m^2^	3–5%	Adults > 65 years old
600 mg/m^2^	8.7%	Pre-existing cardiac disease and hypertension
700 mg/m^2^	18–48%	Combinational chemotherapy

*ATC, anthracyclines*.

### Characteristics of Dox Cardiotoxicity

**Figure 4:**
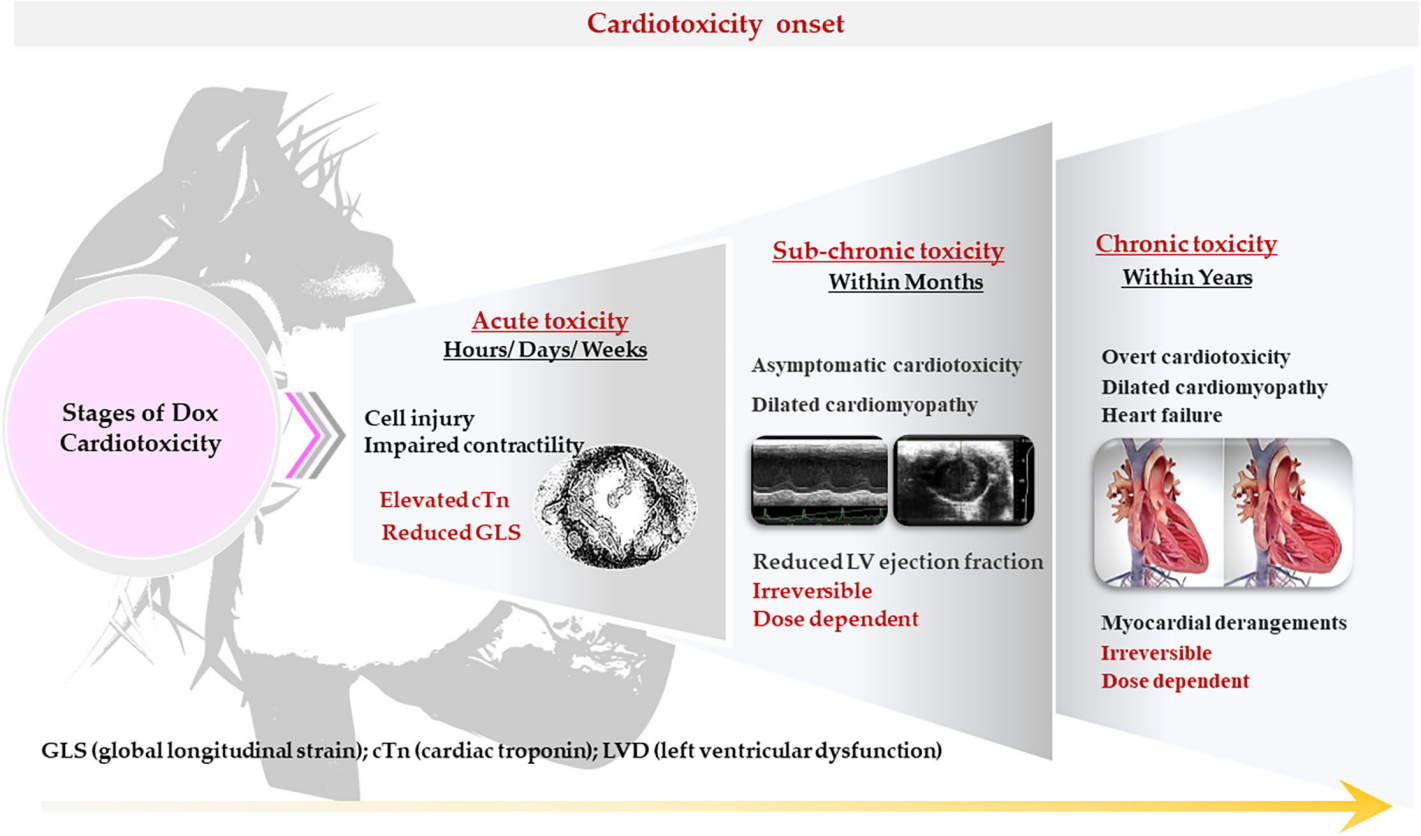
Characteristics of doxorubicin (Dox)– induced cardiotoxicity. The cardiotoxicity in cancer patients is classified according to the time of onset, severity, and characteristics. It may manifest as either acute, sub-chronic (early onset) or chronic (late stage).

The cardiotoxicity in cancer patients is classified according to the time of onset, severity and characteristic, and may manifest as either acute, sub-chronic (early onset) or chronic (late stage) (Table 2). Often, these patients present with subclinical ventricular dysfunction leading to severe cardiomyopathy and eventually myocardial failure (23). Acute cardiotoxicity, which is the first type and is considered rare, manifests after a single dose or course of chemotherapy, and presents with reversible cardiac impairments during or immediately after treatment cessation administration (25). Sub-chronic cardiotoxicity, which is the second type and the principal form of cardiotoxicity, manifests within a year of chemotherapy and presents with irreversible dilated-hypokinetic cardiomyopathy leaning toward cardiac failure (8). The last and perhaps causing the highest burden is chronic cardiotoxicity, emerging years to decades after the last administered dose of Dox and causes irreversible left ventricular dysfunction (LVD) and eventual heart failure (HF) (Figure 4) (7). On the contrary, Cardinale et al. (8) argued that classifying AIC into different categorizes might be primitive and biased, as these classifications were formulated in the 1980s around retrospective studies based on pediatric cancer survivors and their predisposition to cardiomyopathies (8). The authors explained that instead of being different entities, occurring at different times, AIC may be a continuous phenomenon that progresses from myocardial cellular injury to cardiac deformities which progress into asymptomatic cardiotoxicity, and eventually lead to overt HF. This view is supported by reports of increased cardiac troponin (cTn) levels with a concomitant reduction in global longitudinal strain (GLS) soon after the first administered dose of Dox. Beyond their ability to aid in diagnosing myocardial infarction, cTn often precede DIC and are commonly detectable in HF. Additionally, the assessment of GLS is more sensitive to LVD and a better predictor of cardiovascular outcomes when compared to LV ejection fraction (LVEF) (26). Therefore, it is not implausible that AIC might develop as early as after the first administered dose of Dox with clinical symptoms only being detected years after treatment cessation.

**Table 2:** Characteristics of DIC.

**Type**	**Onset**	**Clinical features**
Acute cardiotoxicity	During or immediately after chemotherapy Reversible	Cardiomyocyte injury Depression of myocardial contractility
Sub-chronic cardiotoxicity	Within 1 year after treatment cessation Irreversible Dose dependent	Asymptomatic cardiotoxicity Dilated cardiomyopathy
Chronic cardiotoxicity	More than a year after treatment cessation Irreversible Dose dependent	Overt cardiotoxicity Dilated cardiomyopathy Heart failure

### Mechanism of Dox-Induced Cardiotoxicity

Despite the extensive literature that is currently available on the pathophysiology of DIC, the exact mechanism by which Dox inflicts its adverse reactions remains inconclusive. Nonetheless, accumulating evidence suggests that the activation of cell death pathways during Dox administration may be the primary cause of DIC (27–29). These pathways are mediated by several biochemical processes namely, oxidative stress, inflammation, autophagy, DNA and mitochondrial damage (13, 29–32). The biochemical stimulation of these processes can be traced back to the pharmacology of Dox and its inability to distinguish between normal and cancerous cells, which allows metabolites such as Doxol, DSQ and Dox aglycones to accumulate in the myocardium whereby they inflict their adverse reactions (19, 20, 33). Considering cellular biology, several enzymes like NADPH oxidases (NOXs), NOSs, XOs and peroxisomes, which are located in the sarcoplasmic reticulum, mitochondria, and cytoplasm, account for a significant amount of ROS production (34–36). In the cardiac muscle, mitochondria-induced ROS production is driven by the reduction of Dox to DSQ, *via* NOXs. The infiltration of DSQ in the mitochondria disrupts the electron transport system (ETS), whereby DSQ displaces the antioxidant coenzyme Q10 to accept electrons from complex I and II and then donates them to molecular oxygen instead of transferring the electrons to complex III. This triggers the production of ${\text{O}}_{2}^{-}$, •OH and H_2_O_2_ (34). Additionally, the high affinity of DSQ to cardiolipin allows for its accumulation in the mitochondria resulting in excessive ROS production. By disrupting the ETS, which impairs bioenergetics and induces oxidative stress, DSQ are able to induce mitochondrial toxicity (34), thereby activating the intrinsic apoptotic pathway *via* the cytosolic translocation of caspase 3 to the nucleus (37). In addition, cardiac iron-overload, which is mediated by the enhanced expression of transferrin, an iron transferring glycoprotein, further accelerates Dox-induced oxidative stress by suppressing the activity of endogenous antioxidants [catalase (CAT), superoxide dismutase (SOD) and glutathione peroxidases (GPXs)] which, consequently causes the peroxidation and rupture of membrane lipids and resultant ferroptosis (28). Much like cancer cells, which express Top IIα, cardiomyocytes also express nuclear and mitochondrial Top II, but in the β isoform. This makes the myocardium a suitable target of Dox toxicity, as Dox inhibits Top IIβ activity, to induce apoptosis *via* DNA damage (38). Dysregulated apoptosis is recognized as a necessary step for the onset of left ventricular (LV) remodeling, which is a hallmark of DIC. Another fundamental aspect to LV dysfunction is impaired inflammatory and autophagic response during Dox administration, which exacerbates myocardial cell death *via* the induction of pyroptosis and necroptosis, respectively (28, 39). The induction of Dox pyroptosis is mediated by the upregulation of interleukin 1β (IL-1β) and IL-18 in the presence of cytotoxic N-terminal of gasdermin D proteins, which are activated by NOD-like receptor family pyrin domain-containing 3 (NLRP3) inflammasomes and caspases (1, 3, 4 and 11) (28). Contrary to the aforementioned biochemical processes, the effect of Dox on autophagy is controversial. This is because Dox either suppresses autophagy, resulting in the accumulation of damaged organelles which trigger oxidative stress and inflammation, or increases autophagic response to accelerate the removal of useful cellular components *via* apoptosis (13, 32, 40). Similarly, the upregulation of autophagy markers like light chain 3B (LC3B) has been previously shown to directly interact with receptor-interacting protein-1 (RIPK1) and RIPK3, which promote the formation of necrosomes in the presence of death receptors, such as Fas and tumor necrosis factor receptor 1 (TNFR1), thereby inducing necroptosis (28, 41, 42).

### Prevention of DIC: Conventional Therapy and Limiting Dox Exposure

**Figure 5:**
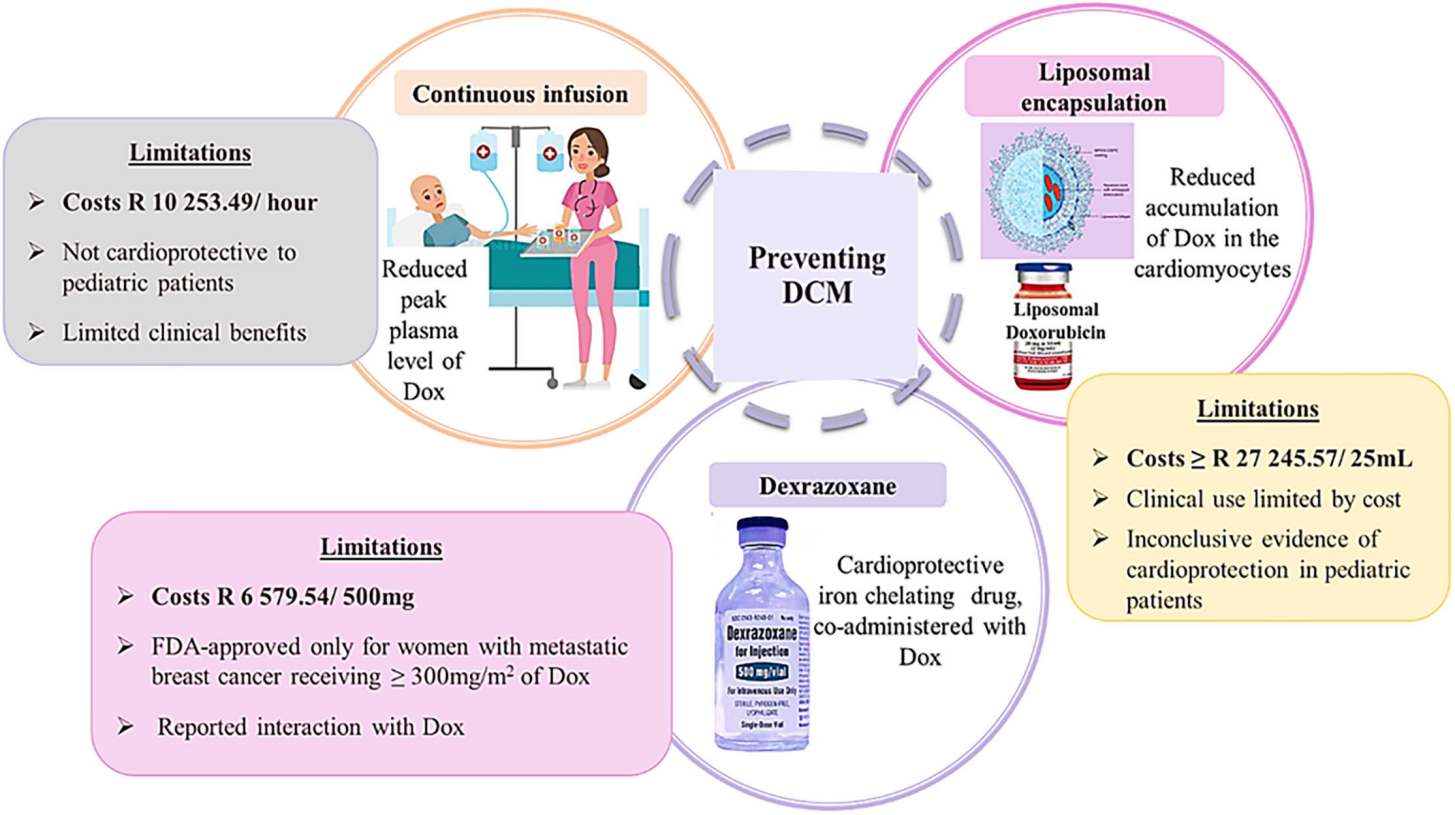
Schematic presentation of preventative strategies of doxorubicin-induced cardiomyopathy. Image adapted from dreamstine.com.

Often, cancer patients who are in complete remission lead very normal and healthy lives until they experience cardiovascular-related abnormalities, such as dyspnea and angina (20). Presumably, when this happens, irreversible signs of cardiotoxicity would have already manifested in these patients. In contrast, Cardinale et al. (8) challenged the irreversibility of AIC, arguing that close monitoring of cancer survivors could allow for early diagnosis and timely treatment initiation, which would likely reverse the cardiotoxicity and therefore, prevent the onset of DIC. The latter can also be mediated by two distinct approaches (Figure 5). The first approach entails the liposomal encapsulation and continuous infusion of Dox with the intension of reducing the plasma levels of Dox and its accumulation in the cardiomyocytes (2). The second approach involves the co-administrative use of Dox with dexrazoxane, which is the only U.S. Food and Drug Administration (FDA) approved cardioprotective drug used in chemotherapy.

#### Liposomal Encapsulation

Briefly, liposomes are miniature spheres that are spontaneously formed by singular or multiple hydrated phospholipid bilayers containing polar groups, which are oriented onto the inner and outer aqueous phase (43). The unique structure of liposomes allows for the encapsulation of bioactive amphipathic, lipophilic, and hydrophilic compounds within its aqueous or lipid compartments to improve drug efficacy. Contrary to standard Dox, liposomal encapsulation of Dox alters its pharmacokinetics. Literature demonstrates that liposomal Dox has a reduced plasma clearance rate which allows for much higher drug concentrations to be present in cancerous tissues than in normal tissues (44). This is because liposomal Dox effortlessly pierces through tumor vasculature, which is highly susceptible to penetration when compared to healthy tissue. With this in mind, the anticancer effect of Dox is preserved while the risk of developing cardiotoxicity is diminished (2). Unfortunately, the excessively high costs of liposomal Dox, which is currently priced at $1,727.18–2,480.54, for a 25-milliliter vial, has drastically limited its use, especially in developing countries. Another limitation is the selectivity of liposomal Dox which is currently FDA approved only for ovarian cancer, multiple myeloma and acquired immune deficiency syndrome–related Kaposi sarcoma (2). These apparent limitations along with the lack of long-term follow-up studies and the inconclusive evidence that exists on the efficacy of liposomal Dox in pediatric cancer patients has further limited its clinical application (45).

#### Continuous Infusion

As another preventative measure, administering Dox in divided doses has been shown to cause significantly lower adverse effects in cancer patients than those receiving bolus infusions of Dox (2, 46). The rationale is that consecutive daily doses of Dox builds patient tolerance and conditions the heart to be less susceptible to DIC, whereas single rapid infusions result in much higher Dox concentrations in the myocardium leading to more severe clinical cardiotoxicity in adult patients (2). In reality though pediatric cancer patients receiving continuous infusions have been shown to have no preserved or improved cardiovascular function when compared to children receiving bolus Dox doses (47). Instead, both participants presented with signs of deteriorating cardiovascular function at the 8-year follow up after the last dose of Dox had been administered, indicating that the continuous infusion had minimal preventative benefits (47). These disparities in treatment response, between adult and pediatric cancer patients, have since discredited the notion that administering Dox in continuous infusions can be considered as a preventative strategy.

#### Dexrazoxane: The Only FDA Approved Cardioprotective Drug

Currently, the only FDA approved cardioprotective drug and most reliable preventative option of DIC is the co-administrative use of Dox with Dexrazoxane (Dex). Briefly, Dex is an iron chelating agent that scavenges the pro-oxidants formed by Dox which drive oxidative stress and mitochondrial dysfunction (13, 48). Additionally, Dex alters the configuration of topoisomerase II beta (Top IIβ), to a closed-clamp structure by tightly binding to the ATP-binding sites of the topoisomerases. This inhibits the binding of Dox to Top IIβ thereby, preventing DNA damage and apoptosis. The cardioprotective benefits of this iron-chelating agent have been determined to be non-selective as the efficacy of Dex transcends numerous malignancies occurring in both adult and pediatric cancer patients (38, 49, 50). Evidently, in a clinical trial of advanced breast cancer, patients that were co-treated with Dex presented with significantly improved left ventricular ejection fraction (LVEF) when compared to patients receiving Dox alone (51). Similarly, Dex prevented AIC in pediatric cancer patients after a 5-year follow-up, where the mean LV fractional shortening and end-systolic dimension Z scores were determined to be noticeably better than those measured in children who had received Dox alone (49). This protection was further highlighted in Dex's ability to preserve the LV wall thickness and thickness-to-dimension ratio in cancer patients after 5-years of treatment cessation (49).

Although truly remarkable, the co-administrative use of Dex has been surrounded by considerable controversy. For instance, Dex is only FDA approved for females with metastatic breast cancer requiring an additional infusion of Dox to regulate tumor progression and eradicate the cancer, after they have received at least 300 mg/m^2^ of chemotherapy (2). Furthermore, two clinical trials, on adult cancer patients, demonstrated an increased risk in the development of secondary malignant neoplasms when Dex was used (52, 53). Subsequently, in Europe and other jurisdictions, a ban in the use of Dex as a cardioprotectant in children was enforced to reduce the risk of developing secondary malignancies in these patients (52). While these claims have since been disputed, the American Society of Clinical Oncology (ASCO) guideline cautioned against the use of Dex in pediatric cancer patients due to the lack of conclusive evidence associated with the use of Dex (52). Another limitation of this drug is claiming that Dex offers greater cardioprotection to females than their male counterparts (2). Therefore, these limitations along with the high costs of Dex, which further limits its use in poorer communities, strongly advocates for the investigation of alternative therapies.

#### Other Cardiovascular Agents

##### Angiotensin Converting Enzyme Inhibitors

Although not FDA approved to be used concurrently with ATCs, several other cardioprotectants, aside from Dex, have been identified to have therapeutic benefits that may aid in alleviating the burden of DIC (23, 54). For starters angiotensin converting enzyme inhibitors (ACEI), which are historically known for their anti-hypertensive properties, are reported to mitigate heart failure by reducing cardiac afterload and systolic wall stress, decreasing aldosterone-induced fibrosis and apoptosis, whilst improving ventricular geometry (55, 56). These drugs are further said to curb the mortality rate in patients with asymptomatic LV dysfunction (55), which makes them suitable therapeutic options for the treatment of DIC. Indeed, a clinical study mimicking the prevention of chronic-cardiotoxicity revealed a gradual deterioration in cardiac function over a period of 12 months, as measured by an LVEF of 48% (56). However, the administration of enalapril, an ACEI, protected against myocardial damage by preserving LVEF, which was found to be 62% at the end of the study when compared to the 61.9% measured at baseline (56). In another study of acute-cardiotoxicity, cancer patients scheduled to undergo chemotherapy were co-treated with valsartan, which is an angiotensin receptor blocker (ARB), for 7 days with the aim (57). Findings from this study revealed the therapeutic ability of valsartan to improve ventricular function, which was indicated by a reduction in the serum levels of brain natriuretic peptide (BNP) and, LV end-diastolic diameter of the left ventricle (LVEDD) and corrected QT dispersion (QTcD) (57). However, although demonstrating promising prophylactic benefits against DIC, ACEI and ARB do not offer complete protection against DIC, but instead lowers the incidence of heart failure and premature death in cancer patients (58). Another area of concern is the high-cost-benefit ratio of administering ACEI (8), which may result in the inaccessibility of the drug to individuals from impoverished backgrounds.

##### Statins

Statins are best known for their ability to reduce low-density lipoprotein (LDL) cholesterol to aid in reversing atherosclerotic plaques, which alleviates the burden of coronary artery disease (59). In the context of DIC, the cardioprotective benefits of statins are associated with the drugs pleiotropic effects, which include their antioxidant and anti-inflammatory properties, as well as their ability to enhance endothelial function (8). This is especially important as one of the primary mechanisms of DIC involves the onset of cardiac oxidative damage (34, 38). In a mice model of DIC, atorvastatin ameliorated Dox-induced oxidative stress and DNA damage, which led to improved myocardial structural integrity (60). Similarly, LV systolic and diastolic function were significantly enhanced in rats co-treated with Dox plus rosuvastatin 4 weeks after treatment cessation (61). In newly diagnosed breast cancer patients, an observational clinical cohort study of, revealed that individuals that were co-treated with ATCs and statins had a lower risk of developing heart failure (HF) than patients that were only treated with ATCs (62). Lastly, pre-treatment with fluvastatin, in an acute model of DIC, demonstrated a significant reduction in oxidative stress, inflammation and apoptosis in the cardiac muscle (63). While these findings clearly highlight the beneficial properties of statins in cardiovascular health, it remains obscure whether these benefits can be sustained over a pro-longed period. Therefore, long-term studies are still needed to establish the long-term effects of statins in these patients.

##### Beta-Blockers

Literature also reports that the therapeutic properties exhibited by beta-blockers (β-blockers) may aid in alleviating the clinical burden of DIC (8, 58). Concisely, β-blockers have been FDA approved for the treatment of several CVDs and their comorbidities such as; hypertension, coronary artery disease, arrhythmias, myocardial infarction and congestive heart failure, just to name a few (64). A clinical study reported that carvedilol, a non-cardio selective β-blocker, prevents ventricular dysfunction in cancer patients receiving ATC treatment (65), while another demonstrated a significant reduction in myocardial strain impairments and troponin levels (66). In another clinical study involving HER2-negative breast cancer patients, receiving combinational chemotherapy including Dox, reported no apparent changes in LVEF and B-type natriuretic peptide between the placebo group and patients that had been treated co-treated with carvedilol (67). This study did, however, report a reduction in troponin I levels which they correlated to the reduced incidence of diastolic dysfunction in carvedilol treated patients (67). In another clinical study, the use of a selective β-blocker, nebivolol administered a week prior chemotherapy induction, preserved LV end-systolic (LVESD) and end-diastolic diameters (LVEDD) and serum levels of N-terminal (NT)-pro brain natriuretic peptide (NT-proBNP) (68). While nebivolol was also found to sustain a 63% LVEF, the reduction in LVEF to 57% was still within normal range and not an indicator of cardiac dysfunction (68). In this study, cardiotoxicity was represented by the significant increase in proBNP levels in the placebo group (68). Seicean and colleagues revealed that the continuation of β-blockers, months after chemotherapy cessation, had a greater therapeutic outcome than administering β-blockers for only the duration of the chemotherapy cycles (69). This outcome was represented by the reduced HF incidence and new HF events (69). However, it appears that the usefulness of β-blockers against chemotherapy-related cardiotoxicity is controversial. For instance, an *in vitro* experimental study revealed the inability of metoprolol to prevent cardiotoxicity in C57Bl6 mice treated with Dox and trastuzumab (70). Similarly, Avila et al. (66) demonstrated carvedilol's inability to mitigate ATC-induced chronic cardiotoxicity in breast cancer patients (67).

### Alternative Medicine: The Efficacy and Adverse Effects of Plant-Based Treatment

In recent decades a growing interest in alternative therapies, consisting of herbal extracts and plant-derived compounds, have been identified as a promising solution to combatting the burden of diseases like cancer and its associated side-effects namely cardiotoxicity, hepatotoxicity and nephrotoxicity, just to name a few (71–74). It is, therefore, no surprise that over 5,000 studies have investigated the therapeutic benefits of herbal-based treatments against DIC. To date, an excessive amount of research has been conducted on flavonoids to illuminate their pharmaceutical benefits as cardioprotective agents. Concisely, flavonoids are secondary metabolites of plants and have been shown to have anti-tumor and anti-oxidation properties in addition to improving cardiovascular outcomes by alleviating endothelial dysfunction and atherosclerosis (75–77). Accumulating evidence suggests that flavonoids can be very effective in attenuating DIC (78–80). For example, Apigenin, which is a non-mutagenic plant flavone, alleviated Dox-induced myocardial damage, in male rats, by preserving the hearts structural integrity and by improving its ejection fraction, fractional shortening, LV internal diameter end in diastole (LVIDd) and LVID end in systole (LVIDs) (81). These effects were associated with the flavonoid's ability to scavenge lipid peroxides through enhanced myocardial superoxide dismutase (SOD) activity and glutathione (GSH) content. Subsequently, a decrease in Dox-induced apoptosis *via* the reduction of BAX and Caspase-3 activity, and enhanced Bcl-2 expression, was observed (81). The reported benefits were further confirmed by the apparent reduction of myocardial injury biomarkers [cTn, lactate dehydrogenase (LDH) and creatine kinase-MB (CK-MB)], which explained the improved cardiac function in these animals.

Additionally, Latifolin, which is one of the major flavonoids found in *lignum dalbergiae odoriferae* and known for its anti-inflammatory and cardioprotective properties, was recently reported to have therapeutic benefits against DIC (80). In this study, Latifolin mitigated myocardial injury by decreasing macrophage expression of M1 [i.e., inducible nitric oxide synthase (iNOS) and Cluster of Differentiation 86 (CD86)] and M2 [i.e., CD206, interleukin-10 (IL-10) and IL-4R] polarization in Dox treated animals. As a result, a significant increase in LVEF and LV fractional shortening (LVFS), with a concomitant reduction in LDH levels, was observed (80). Similarly, Luteolin, a common flavonoid existing in numerous plants, mitigated Dox-induced cardiomyocyte contractile dysfunction by enhancing the cells peak shortening amplitude and maximal velocity of re-lengthening and shortening (79). Luteolin, further attenuated cardiomyocyte injury by preserving mitochondrial membrane potential and autophagy, *via* the Drp1/mTOR/TFEB signaling pathway, as well as preventing mitochondrial-induced ROS activity and apoptosis. While these flavonoids have proven to be highly effective at preventing or alleviating the burden of DIC, it remains unknown what effect these flavonoids will have on the anti-carcinogenic properties of Dox. Evidently, this is a major concern in cardio-oncology research as novel therapeutic agent may potentiate the progression of cancer by inhibiting the efficacy of chemotherapeutic agents. The fact that flavonoids naturally possess high antioxidant properties could potentially benefit the cancer cells, as they may redirect some of these antioxidants to enhance the activity of their own antioxidants thereby preventing Dox-induced oxidative stress and apoptosis, which may potentiate the cancer. For this reason, it is not only important that the search for novel cardioprotective agents continues, but that their effect in cancer models be investigated, especially when used with other chemotherapeutic drugs.

#### Cardioprotective Potential of Quercetin Against DIC

**Figure 6:**
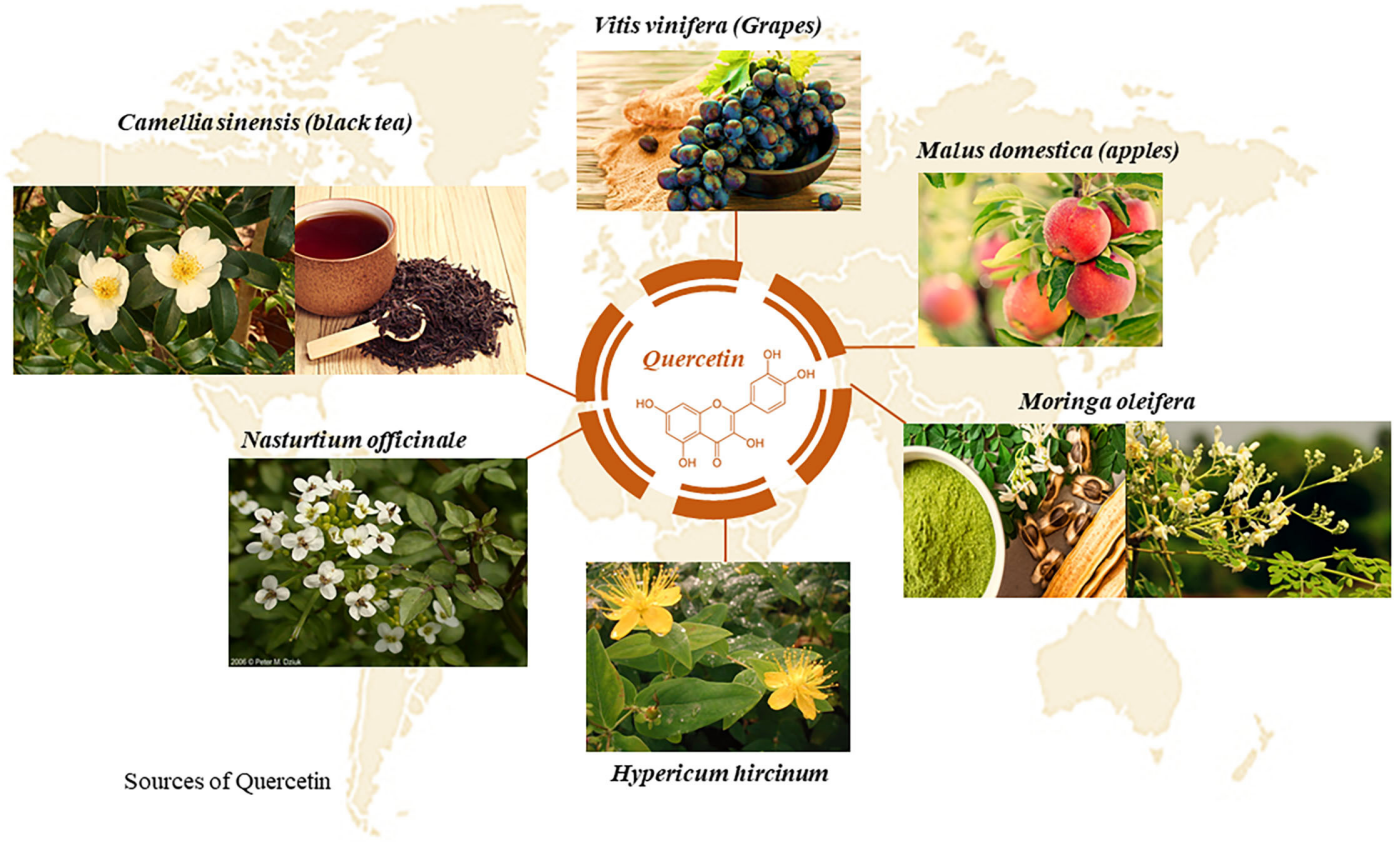
Plant sources of quercetin, a bioflavonoid with numerous medicinal properties.

Quercetin is an important bioflavonoid, belonging to the class of flavanols, found in numerous plants and plant products such as, *Camellia sinensis*, grapes and *Nasturtium officinale* (Figure 6) (82). The therapeutic benefits of quercetin are primarily attributed to its anti-inflammatory, antioxidative, anti-proliferative and anti-histamine properties (83). These pharmacological benefits have been reported in experimental models of cardiovascular disease, hepatopathy and anti-cancer studies (84–86). Previously, Chen and colleagues (82) hypothesized that the cardioprotective properties of quercetin were driven by its effect on mitochondrial function *via* the activity of 14-3-3γ, a protein belonging to the highly conserved multifunctional 30 kDa acidic protein family. In this study, quercetin mitigated DIC by enhancing the expression of 14-3-3γ. This was demonstrated by an increase in the levels of endogenous antioxidants, GSH, SOD, CAT and GPx, in the cardiomyocytes (84). Subsequently, the cardiac cells were protected from Dox-induced oxidative damage, as shown by the significant reduction in lactate dehydrogenase (LDH), lipid peroxidation and ROS activity. Additionally, quercetin decreased the activity of the apoptotic markers, caspase 3 activity, mitochondrial permeability transition pore and annexin v and propidium iodide (84). In another study, quercetin was shown to preserve the structural integrity of the cardiomyocytes by decreasing Dox-induced expression of proteins involved in modulating protein folding (83). The downregulation of these proteins led to a reduction in ROS activity which reduced the degree of incorrectly folded proteins thereby, attenuating the expression of 60 kDa heat shock protein and heat shock protein beta-1, alpha-crystallin B, stress-induced-phosphoprotein 1 and T-complex protein 1 (83). Dong et al. (87) then showed a significant reduction in DNA damage and mitochondrial ROS production following the co-administrative use of Dox with quercetin. These findings were supported by another report demonstrating how quercetin mitigates Dox-induced myocardial dysfunction by improving LVEF, LVFS, LVEDD and LVESD in C57/BL6 mice (87). Consequently, an increased survival outcome in the quercetin plus Dox treated mice vs. those treated with Dox alone was observed (87). While the cardioprotective benefits of quercetin are undeniable and transcend DIC, it is imperative that we establish the effect of quercetin on cancer cells and its effect on the anti-carcinogenic properties of Dox.

#### Therapeutic Benefits of Quercetin Against Cancer

A study by Wang et al. (85) assessed the anti-cancer properties of quercetin on human hepatocellular carcinoma cells HepG2 and Hep3B, triple negative MDA-MB-231 breast cancer cells, and colorectal cancer cells HCT116. In this study, quercetin was shown to trigger autophagy in the cancer cells by increasing the expression of autophagy markers, ATG7, LC3-II and p62 (85). These findings promoted lysosomal activation and nuclear translocation of transcription factor EB in the cancer cells, which enhanced quercetin-induced cell death, independent of p53 expression. The former was attributed to the degradation of ferritin light chain (FTL) and ferritin heavy chain (FTH), and the induction of lipid peroxidation, which resulted in cancer cell toxicity (85). These finding are especially important as the release of iron from ferritin storage has been shown to cause iron-overload, which may trigger the activation of tumor suppressor genes initiating ferroptosis (88). Additionally, quercetin also enhanced the expression of the pro-apoptotic proteins, Bid, cytochrome C expression in the cytoplasm and cleavage of caspase 9, which accelerated cancer cell death *via* apoptosis (85). The anti-cancer properties of quercetin have also been reported in tumor bearing mice, mimicking a breast cancer model (89). Here, quercetin stimulated rapid tumor regression and significantly increased animal survival when compared to the untreated mice (89). Similarly, the anti-carcinogenic properties of quercetin also prevented angiogenesis *via* enhanced expression of thrombospondin-1, which is an endogenous anti-angiogenic factor protein that inhibits tumorigenesis (90).

#### The Effect of Quercetin on the Pharmacokinetic Profile of Dox

In the context of co-administering Dox with quercetin, data obtained from previous studies and PubChem indicate that quercetin is a strong inhibitor of CYP2D6 (0.65 ± 0.13 μM) and CYP3A4 (5.5 ± 0.7 μM) (91, 92). The inhibitory effect of quercetin on CYP3A4 (1.97 μM) was also confirmed by Choi et al. (21), who additionally showed an inhibition of P-glycoprotein (P-gp), a Dox transporter, in rats and MCF-7/ADR cells that were co-treated with Dox plus quercetin. As a general rule, drugs that are inhibitors or substrates of the same drug should not be administered simultaneously. However, considering that Dox is a known potent inhibitor of CYP2D6 and a substrate of CYP3A4, these findings suggest that quercetin may influence the pharmacokinetic profile of Dox. It is well-documented that the metabolism of Dox to its secondary metabolites is facilitated by the induction of CYP3A4, which consequently increases the accumulation of its cardiotoxic metabolites in the myocardium (33). Therefore, the inhibition of CYP3A4, as well as other metabolizing enzymes, could be an alternative therapeutic target that may aid in alleviating the burden of DIC and further explains the cardioprotective benefits of quercetin against DIC. The former may also aid in enhancing the efficacy of Dox through increased plasma levels of the unmetabolized Dox in the absence of CYP3A4 and CYP2D6 activity. Indeed, Choi et al. (21) revealed that co-administering Dox with quercetin enhanced the peak plasma concentrations and bioavailability of Dox. This increase was attributed to the inhibition of P-gp which decreased phase I metabolism of Dox resulting in an increased absorption of Dox in the gastrointestinal tract (21). Therefore, these findings strongly suggest that the use of quercetin as a cardioprotective alternative against DIC is unlikely to reduce the chemotherapeutic benefits of Dox.

#### Pinocembrin, a Diversely Therapeutic Flavonoid, Attenuates DIC

**Figure 7:**
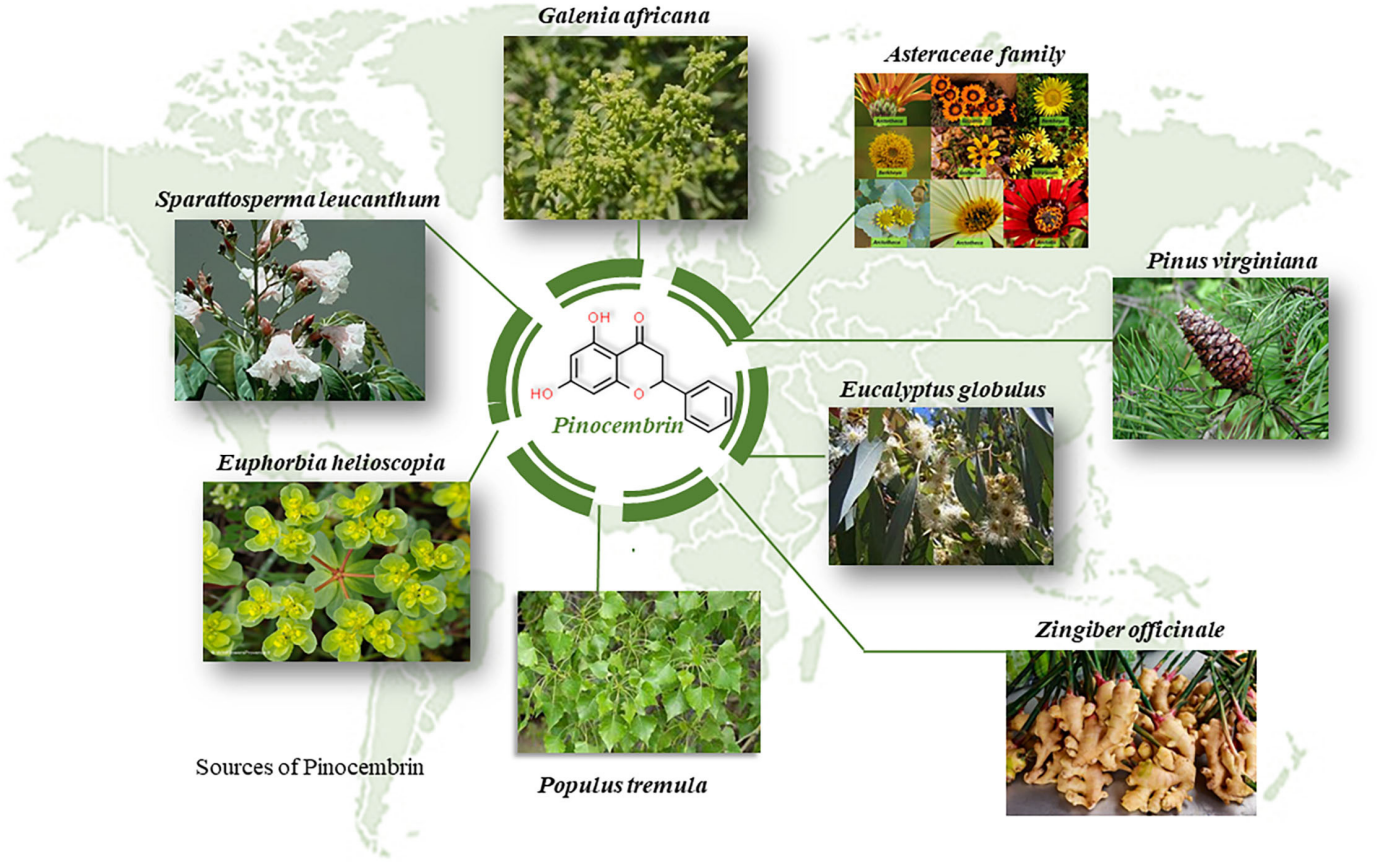
Plant sources of pinocembrin all over the globe. Pinocembrin is a plant derived flavonoid with numerous therapeutic benefits.

Another flavonoid of interest is pinocembrin (Pin), which possesses potent cardioprotective benefits against DIC (93). Briefly, Pin is a pharmacologically active flavonoid found abundantly in propolis and may also be isolated in numerous plants such as *Galenia africana* and *Asteraceae* families, to name just a few (94, 95) (Figure 7). Our laboratory was the first to investigate the co-administrative effects of Pin with Dox in an *in vitro* cardiomyocyte and cancer cell model. Since Dox accumulates in cardiac mitochondria, it disrupts the transfer of electrons across the electron transport chain (ETC) where it re-directs the electrons to generate ROS and trigger mitochondrial outer membrane permeabilization (MOMP) whilst impairing mitochondrial bioenergetics (13). Generally, MOMP is considered an irreversible process that drives end-stage cell death, such as apoptosis and necrosis, *via* the activation of caspases and autophagy, by diffusing the presence of several proteins that are usually situated between the outer (OMM) and inner (IMM) mitochondrial membranes inside the cytosol. In our study, we mimicked an *in vitro* model of chronic DIC by exposing cardiomyocytes to Dox for 6 days. We then showed that Pin, as an adjunct to Dox, alleviated mitochondrial-induced ROS and lipid peroxidation by enhancing the antioxidant capacity (GSH and SOD) of the cardiomyocytes when compared to cells that were treated with Dox alone. Consequently, cardiac mitochondrial function was significantly ameliorated after co-treatment with Pin, as could be seen by increased mitochondrial flux ratios, ATP-linked respiration, ATP turnover and maximal respiration in the cells, as well as an increase in cells' spare respiratory capacity. With this improvement, the cardiomyocytes mitochondrial membrane integrity was preserved, which led to a noticeable reduction in caspase 3/7 activity and resultant apoptosis (93).

These findings were recently confirmed in an *in vivo* study conducted by Gu et al. (96). In this study, co-treatment with Pin attenuated Dox-induced cardiac dysfunction by improving the LVEF, LVFS, LVIDd and LVIDs of male C57BL/6 mice. Likewise, co-treatment with Pin decreased myocardial fibrosis and injury, as determined by histological analysis and reduced serum LDH and CK-MB levels (96). Since Dox triggers numerous cell death pathways, other than apoptosis, the authors studied the effect of Pin on Dox-induced pyroptosis, which is initiated by an impaired inflammatory response. As a co-treatment, Pin attenuated pyroptosis-mediated cell death by reducing caspase-1 activity, protein and serum expression of the inflammatory cytokines, IL-1β and IL-16, as well as inflammasomes, NOD-like receptor protein 3 (NLRP3) and gasdermin-D (GSDMD). This reduction could be attributed to Pin's ability to activate the Nrf2/ Sirtuin 3 (Sirt3) pathway, which suppresses cell death and DIC (96).

#### The Effect of Pinocembrin on the Efficacy of Doxorubicin as a Chemotherapeutic

While alternative therapies have proven to be quite effective at protecting against the onset and progression of DIC in *in vitro* and *in vivo* cardiac experimental models, most of these therapies have not been investigated in cancer models to determine their effect on the anti-carcinogenic properties of chemotherapeutic drugs. The latter is an ongoing problem in cardio-oncology research as most plant-derived cardioprotective agents have high antioxidant and anti-apoptotic properties, which could benefit cancer cells by boosting their endogenous antioxidant levels and in turn, protects them against Dox-induced cytotoxicity. This outcome would be quite detrimental, especially for cancer patients, as this would not only potentiate cancer progression but, could very well increase cancer-related mortalities. For this reason, it is crucial that when investigating novel cardioprotective agents, in models of DIC, the risks associated with these agents be simultaneously assessed in cancer models to ensure that the efficacy of chemotherapeutic drugs is preserved and not inhibited by new cardioprotectants (97). In this context, our group investigated the efficacy of Dox when used in combination with Pin in human estrogen receptor positive breast cancer cells (93). In this study, Pin as an adjunct to Dox, had no significant effect on the antioxidant profile of breast cancer cells which was demonstrated by the comparable GSH and GSSG levels between these cells and those treated with Dox alone. In this way Dox was still able to induce oxidative stress by channeling electrons away from the ETS which was confirmed by the observed reduction in the cancer cells metabolic status. Consequently, the efficacy of the mitochondria was compromised, which facilitated Dox-mediated mitochondrial damage and in turn triggered cell death pathways. We further found that while co-treatment with Dox plus Pin induced relatively lower apoptosis when compared to cells treated with Dox alone, Pin, as an adjunct to Dox, led to a significantly higher degree of necrosis in the breast cancer cells. These findings suggest that the co-administration of Dox plus Pin might synergistically aid in eradicating the cancer.

#### The Potential Effect of Pinocembrin on the Pharmacokinetic Profile of Dox

While only two studies have reported on the prophylactic benefits of Pin against DIC, the extensive pharmacological properties of Pin have led to its approval as a novel therapeutic drug by the Chinese Food and Drug Administration (CFDA), and its safety and pharmaceutical benefits are currently being studied in phase II clinical trials (94). Previous clinical and pre-clinical experimental studies have demonstrated the good pharmacokinetic profile of Pin, which is highlighted by its rapid absorption and wide distribution with negligible residue accumulation (94, 98, 99). In the context of Dox, Pin being a known inhibitor of CYP3A4 and also being implicated in the inhibition of CYP2D6, suggests that co-administering Dox with Pin might give rise to herb-drug interactions (100). Depending on how potently Pin inhibits CYP2D6, in comparison to Dox, may either increase the bioavailability of Dox plasma concentration or reduce it. Since Pin also inhibits CYP3A4 the metabolism of Dox *via* NADPH CYP reductases may be impaired resulting in a reduction in Doxol, semiquinones and aglycones in the circulatory system, and in this manner reduce the severity of cardiotoxicity. Indeed the use of Pin as an adjunct to Dox has already been shown to mitigate DIC therefore, the effect of Pin on CYP3A4 potentially explains how Pin influences the pharmacokinetic profile of Dox to offer cardioprotection (93, 96). Furthermore, the reduction in the biotransformation of Dox to its secondary metabolites could also be considered beneficial in eradicating cancer since Dox, in its unmetabolized form, is reported to have a more potent tumoricidal effect than its metabolites, which are suggested to suppress the anti-cancer properties of Dox (33, 101, 102). These observations are in line with our previous results on the MCF-7 breast cancer cells which revealed no significant reduction in the apoptotic effects of Dox when co-administered with Pin. Nonetheless, the validity of these claims still requires further investigation.

#### Risks of Drug-Interactions Between Flavonoids and Chemotherapeutics

**Figure 8:**
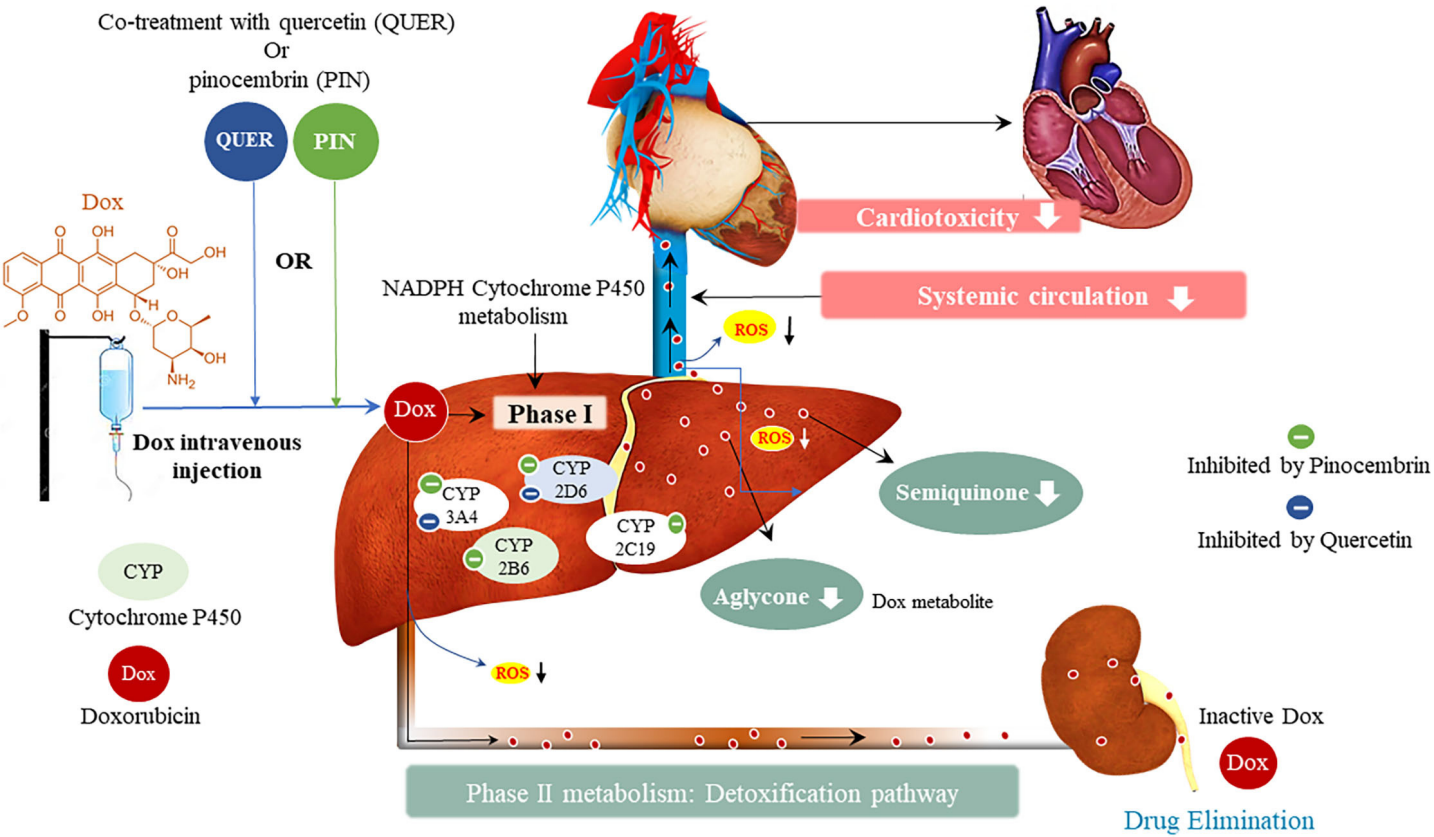
Metabolism of doxorubicin (Dox) and resultant Dox-induced cardiotoxicity, and the potential effect of pinocembrin and quercetin on Dox metabolism. Cytochrome P450 (CYPs) enzymes allow us to predict the pharmacokinetic and pharmacodynamic variability that may exist in novel therapeutics. Doxorubicin inhibits CYP2D6, CYP2C19, and CYP2B6, whilst inducing CYP3A4. The interaction of Dox with these metabolizing enzymes formulates the toxic metabolites semiquinone and aglycone, which induce cardiotoxic side effects.

Considering the characteristics of DIC and the combinational use of novel cardio-protectants with Dox, the risk of inducing drug-drug interactions, that may present with clinically significant reactions, is quite high. This view is supported by the U.S Food and Drug Administration (FDA) who stipulated that due diligence must be done when introducing new therapeutics, by conducting pre-clinical and clinical studies before these drugs are marketed to be used by the public (103). The initial screening of pharmacokinetic profiles of new therapeutics is done by performing *in vitro* experiments using Vivid^®^ recombinant CYP450 enzymes, which measure the activity of drug metabolizing enzymes. This highlights the importance of *in vitro* studies as it is not feasible to study unanticipated drug-drug interactions in human subjects (104). Briefly, CYPs are metabolizing enzymes that drive the phase I metabolism of most drugs and lipophilic xenobiotics, which make them relevant entities in clinical pharmacology (Figure 8) (105). The CYP enzymes, that may be associated with chemotherapy, are categorized into two classes, namely class I and class II. Class I enzymes (CYP1A1, CYP1A2, CYP2E1 and CYP3A4) lack functional polymorphic relevance and are active in the metabolism of pre-carcinogens and other drugs (106). Class II CYPs (CYP2C9, CYP2C19 and CYP2D6) are highly polymorphic and are responsible for the phase I metabolism of various drugs, but not pre-carcinogens (106).

In the context of combinational treatment, using Dox with other CYP2D6 and CYP3A4 inhibitors, has been demonstrated to cause clinically significant interactions, which are likely to enhance Dox plasma concentrations thereby, increasing the severity and incidence of adverse reactions even at lower doses (107, 108). The opposite is also true, the concurrent use of Dox with other CYP2D6 and CYP3A4 inducers, may accelerate drug clearance which would reduce the efficacy of the drug thereby, potentiating the disease state (107). In this view, it is important to note that the drug-drug interactions can have minor, mild or fatal effects depending on the type of inhibition, i.e., reversible or irreversible (109). These type of inhibitions are based on the inactivation of the CYP enzyme *via* metabolic intermediates that bind reversibly or irreversibly to the enzyme. The clinical implications of the irreversible inhibition are expected to last longer than those of the reversible inhibitor after multiple treatment doses (100). This enables clinicians to plan for the appropriate scheduling of sequential regimens that either both inhibit or induce CYP2D6 and CYP3A4. The use of combinational therapy is further supported by the large and flexible active sites of the CYP enzymes, which readily adapt to concurrently accommodate several substrates with distinct structures, without inducing any adverse reactions (100, 109). This might explain how Pin, which is known to cause irreversible CYP3A4 inhibition (100), was able to mitigate Dox-induced cardiotoxicity without reducing the anti-carcinogenic properties of Dox (93).

Although CYP2C19 and CYP2B6 have not been closely associated with Dox, they have been reported to influence cardiovascular outcomes and are involved in the metabolism of other chemotherapeutic agents (106). The inhibition of CYP2C19, in patients with acute coronary syndrome, has been implicated in the occurrence of stent thrombosis and myocardial death (110). In addition, CYP2C19 inhibition in a clinical study involving readmitted patients with myocardial infarction, demonstrated an increased risk of reinfarction (111). In contrast, another clinical study, of patients receiving dual antiplatelet therapy, revealed no significant effect on platelet aggregation following CYP2C19 inhibition (112). In essence these variations in the clinical outcome of CYP2C19 activity highlight the intricacy of pharmacokinetics in treatment and disease response.

## Conclusion

In essence the major issue with the prevalence of DIC is the efficacy of Dox, as an anti-carcinogen. Due to its contribution to the overall improvement in the survival of cancer patients, Dox has been kept in clinical practice whilst the risk of developing cardiovascular dysfunction accumulates. It is, therefore, no surprise that a plethora of work has focused on finding alternative therapies, by using natural compounds like flavonoids, to prevent DIC. Since flavonoids have been continuously reported to mitigate Dox-induced cardiac oxidative damage and cardiomyocyte loss, suggests their suitability as cardioprotective agents against DIC. However, considering the risk of drug interactions, adopting the concept of drug-safety at the initial screening of novel cardioprotectants may aid in preventing unanticipated drug-interactions that may present with clinically significant reactions. In this way, the discovery and development of new alternative therapies, as adjuvants to Dox, can be fast-tracked.

## Author Contributions

NS conceptualized and wrote the manuscript. NS, RJ, SN, LM, BH, KG, DV, and RB edited and approved the final draft of the manuscript. All authors contributed to the article and approved the submitted version.

## Author Disclaimer

The content hereof is the sole responsibility of the authors and do not necessarily represent the official views of the SAMRC or the NRF.

## Conflict of Interest

RB is employed by BioPharm. The remaining authors declare that the research was conducted in the absence of any commercial or financial relationships that could be construed as a potential conflict of interest.

## Publisher's Note

All claims expressed in this article are solely those of the authors and do not necessarily represent those of their affiliated organizations, or those of the publisher, the editors and the reviewers. Any product that may be evaluated in this article, or claim that may be made by its manufacturer, is not guaranteed or endorsed by the publisher.
